# Mathematical Modeling of the Phenomenon of Space-Charge Breakdown in the Galvanostatic Mode in the Section of the Electromembrane Desalination Channel

**DOI:** 10.3390/membranes11110873

**Published:** 2021-11-13

**Authors:** Aminat Uzdenova, Makhamet Urtenov

**Affiliations:** 1 Department of Computer Science and Computational Mathematics, Umar Aliev Karachai-Cherkess State University, 369202 Karachaevsk, Russia; 2 Department of Applied Mathematics, Kuban State University, 350040 Krasnodar, Russia; urtenovmax@mail.ru

**Keywords:** ion-exchange membrane, space charge, galvanostatic mode, desalination, mathematical modeling, Nernst–Planck–Poisson equations

## Abstract

One of the ways to increase the efficiency of the desalination process in membrane systems is to use intensive current modes. Recently, the phenomenon of space-charge breakdown was theoretically described for desalination under intensive current modes. The space-charge breakdown is a decrease in the magnitude and size of the extended space charge regions (SCRs) of opposite signs, formed at the cation- and anion-exchange membranes in the desalination channel, when they approach each other. Therefore, this phenomenon negatively affects the intensity of electroconvection and the efficiency of mass transfer in membrane systems. We report the results of the first theoretical analysis of the space-charge breakdown in the galvanostatic electric mode, which is generally used in the research and operation of membrane systems. For this purpose, a one-dimensional model of the ion transfer of the electrolyte solution in the section of the desalination channel at the direct current is developed. The regularities of changes in the extended SCRs in the galvanostatic mode are determined. A relation is obtained for the onset time of the space-charge breakdown, which makes it possible to determine the parameters of the effective operation of the membrane system.

## 1. Introduction

Membrane systems form the basis of electrodialysis, nano- and microfluidic devices, which are used in water purification, agricultural processing (milk, wine, etc.), chemical analysis and many other human activities [1,2,3,4]. These applications of membrane systems are determined by the selective properties of ion-exchange membranes, which allow ions of the one sign (counterions) to pass through and prevent the movement of ions of the opposite sign (coions) [5]. The difference in the behavior of ions of the opposite signs in the membrane leads to the formation of a space charge region (SCR) at its surface [6].

**Figure 1:**
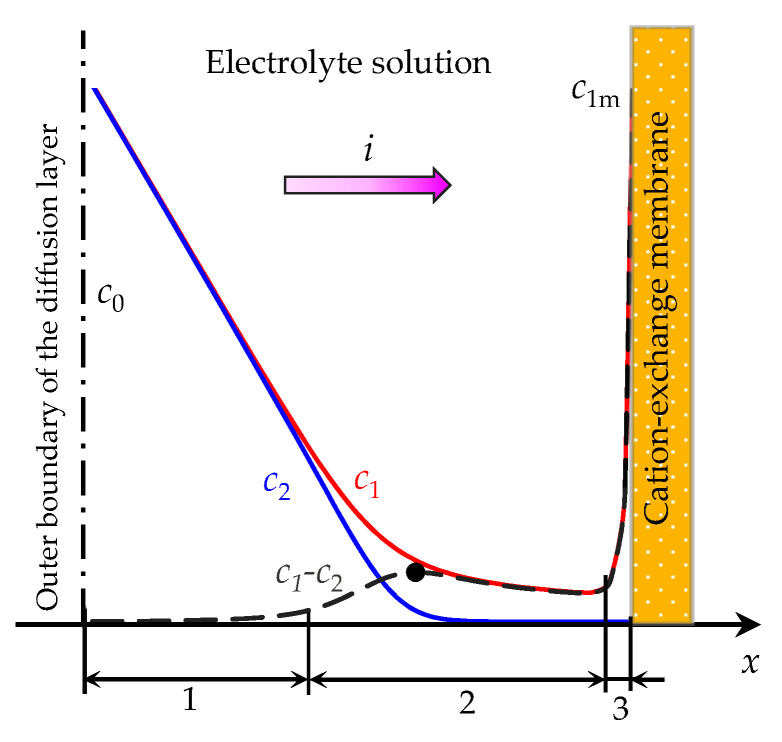
Schematic concentration profiles of cations (*c*_1_, the red line) and anions (*c*_2_, the blue line), the difference in the concentration of positive and negative ions (*c*_1_-*c*_2_, the dashed line) in the diffusion layer adjacent to the surface of a cation-exchange membrane [6]. The current density, *i*, is flowing across the system; the electrolyte concentration in the solution bulk is denoted by *c*_0_; the cation concentration at the solution/membrane interface is denoted by *c*_1m_. The numbers indicate different regions of the diffusion layer: the electroneutral region (1), the extended (2) and the quasi-equilibrium (3) parts of the space charge region (SCR). The black dot denotes the local maximum of the extended SCR.

Rubinstein and Shtilman [6] theoretically described the mass transfer process in the diffusion layer of the selective surface based on the Nernst–Planck and Poisson equations for the electric potential in the one-dimensional (1D) case. The obtained numerical solution at overlimiting currents shows that at the ion-selective surface, the concentration of counterion exceeds the concentration of coion, and an SCR is formed (Figure 1). With an increase in the current density, the minimum concentration of counterion decreases insignificantly, while the thickness of the SCR increases. Therefore, this region is usually called the extended SCR [7]. Thus, the following regions are distinguished in the structure of the diffusion layer at the surface of the ion-exchange membrane: the electroneutral region (indicated by 1 in Figure 1), the extended (2) and quasi-equilibrium (3) parts of the SCR. At a constant potential drop (or current density), a quasi-stationary state is established with time, in which the ion flux from the outer boundary of the diffusion layer is balanced by electromigration and diffusion transfer. In this state, in the electroneutral region, the electromigration flux is equal to the diffusion flux, and, accordingly, to half of the total ion flux from the outer boundary. In the extended SCR, electromigration dominates over diffusion and the diffusion flux can be neglected. Thus, at the overlimiting current, a local maximum of the space charge is formed at the cation-exchange membrane (CEM) (since at the surface of the CEM the space charge is formed by positive cations) and a local minimum at the anion-exchange membrane (AEM) (since at the surface of the AEM the space charge is formed by negative anions).

The emergence of the SCR at the membrane surface explained the nonlinear shape of the current-voltage characteristic (CVC) of the CEM, in which the following regions can be distinguished: the initial linear region, the region of slow current change (plateau), followed by the region of accelerated growth with increasing potential (overlimiting currents) [6,8]. The first region of the CVC corresponds to a state with a rather high ion concentration in the region near the membrane. When an electric current flows through the ion-exchange membrane, the ion concentration decreases on one side of the membrane and increases on the other due to the selective transfer of counterions in the membrane. With the increase in the potential drop, almost complete depletion of ions in the region at the membrane surface in the channel of desalination and the transition of the system to the limiting state are observed [9,10]. In this state, a plateau is observed on the CVC, which describes the saturation of the current corresponding to the almost complete depletion of ions at the membrane surface [11,12]. With a further increase in the applied potential drop, the current takes on values greater than the limiting. For dilute electrolyte solutions, experimental and theoretical studies (a detailed review in [13]) have shown that one of the main mechanisms of overlimiting growth in membrane systems is electroconvection, that is, the entrainment of liquid molecules by hydrated ions (which form a space charge at the ion-selective surface) under the action of the electric force. At overlimiting currents, in addition to the diffusion and electromigration mechanism of the ion delivery to the membrane surface, electroconvective mixing of the solution appears. The formation and modification of the extended SCR determines the intensity of electroconvection. Therefore, to determine the upper limit of the possibilities for increasing mass transfer due to electroconvection, it is of particular interest to change the SCR during the space-charge breakdown.

To simulate desalination with intensive currents, it is necessary to consider the entire desalination channel formed by the AEM and CEM. Liu et al. [14] numerically investigated such a system taking into account electroconvective instability and showed that the operation of the system in this mode leads to a decrease in the mass transfer rate and the formation of a peak on the current-voltage characteristic of the system

Numerical modeling of the mass transfer process in the membrane system until complete desalination of the electrolyte solution was carried out in the article [15]. For this purpose, a 1D model of the desalination of the quiescent electrolyte layer between AEM and CEM under the action of an electric field was built. It was shown that in such a system, over time, the local minimum and maximum of space charges (formed at AEM and CEM) move as single soliton-like waves deep into the solution, towards each other. Then, when the distance between the SCRs of different signs is reduced, they are discharged. A similar phenomenon was also investigated for the 2D case, taking into account the development of electroconvection [16]. This process of decreasing the magnitude and size of the expanded SCRs of opposite signs, formed at the CEM and AEM in the desalination channel, when they approach each other, was called the space-charge breakdown [15,16]. In the case of a membrane system, the breakdown is accompanied by an increase in the charging current and not in the conduction current [15,16]. In the breakdown region, the size and number of electroconvective vortices decrease, which leads to a decrease in the local current density and the efficiency of the desalination process [16]. Thus, the space-charge breakdown phenomenon determines the upper limit of a possible increase in the rate of mass transfer (that is the desalination performance) due to electroconvection [16].

The above-mentioned studies [14,15,16] were carried out for the potentiostatic and potentiodynamic modes, when the potential drop in the system is set, while experimental research and operation of membrane systems generally occurs in galvanostatic (or galvanodynamic) modes [17]. The objective of this work is a theoretical analysis of the basic regularities of the space-charge breakdown on the basis of a mathematical model of the desalination process in the galvanostatic mode. A 1D mathematical model of ion transport in the cross-section of the desalination channel at a constant current density is developed. This model makes it possible to evaluate the effect of the system parameters on changes in the SCRs and the time of the space-charge breakdown, taking into account the selective properties of ion-exchange membranes. A relation is obtained for the onset time of the space-charge breakdown, which makes it possible to determine the parameters of the effective operation of the membrane system.

## 2. Mathematical Model

### 2.1. Governing Equations and Boundary Conditions

Consider a quiescent layer of the binary electrolyte solution located between two ion-exchange membranes: AEM and CEM. The normal to the surface membrane coordinate, *x,* varies from 0 (the solution/AEM interface) to *h* (the solution/CEM interface).

In the 1D case, the mathematical model of the non-stationary ion transfer includes the Nernst–Planck equation, Equation (1); the matter conservation equation (in the absence of chemical reactions), Equation (2); the Poisson equation, Equation (3). Convective transfer is not considered. This equation system for a binary electrolyte is as follows:

(1) $$j_{n}=-\frac{F}{RT}z_{n}D_{n}c_{n}\frac{\partial \phi }{\partial x}-D_{n}\frac{\partial c_{n}}{\partial x},n=1,\;2,$$

(2) $$\frac{\partial c_{n}}{\partial t}=-\frac{\partial j_{n}}{\partial x},\;\;n=1,\;2,$$

(3) $$\varepsilon_{0}\varepsilon_{r}\frac{\partial^{2}\phi }{\partial x^{2}}=-F\left(z_{1}c_{1}+z_{2}c_{2}\right),$$

where $j_{n},c_{n},\;D_{n},z_{n}$ are the flux density, concentration, diffusion coefficient and charge number of ion *n*, respectively; ϕ is the electric potential; *ε_0_* is the dielectric permittivity of vacuum; *ε_r_* is the solution relative permittivity (assumed to be constant); *F* is the Faraday constant; *R* is the gas constant; *T* is the absolute temperature. $j_{1},\;j_{2},\;c_{1},\;c_{2},\;\phi$ are unknown functions of time, *t*, and coordinate, *x*.

The current density in the system, *i*, is equal to the sum of the faradaic (or conduction) current, $i_{F}=F\left(z_{1}j_{1}+z_{2}j_{2}\right),$ and the charging (or displacement) current, $i_{c}=-\varepsilon_{0}\varepsilon_{r}\frac{\partial^{2}\phi }{\partial x\partial t}$, which is associated with the formation and change of the space charge [18,19]:

(4) $$i=i_{F}+i_{c},$$

The boundary conditions for the concentrations of counterion set constant values that are *N_a_* (*N_c_*) times greater than the initial concentration of the electrolyte *c*_0_ [6]:

(5) $$c_{1}\left(h,t\right)=N_{c}c_{0},$$

(6) $$c_{2}\left(0,t\right)=N_{a}c_{0}.$$

The boundary conditions for the concentrations of coion are formulated using the equation of continuity of the ion flux at the solution/membrane interface:

(7) $$\left(-D_{1}\frac{\partial c_{1}}{\partial x}-\frac{F}{RT}z_{1}D_{1}c_{1}\frac{\partial \phi }{\partial x}\right)\left(0,t\right)=\frac{T_{1A}}{Fz_{1}}i,$$

(8) $$\left(-D_{2}\frac{\partial c_{2}}{\partial x}-\frac{F}{RT}z_{2}D_{2}c_{2}\frac{\partial \phi }{\partial x}\right)\left(h,t\right)=\frac{T_{2C}}{Fz_{2}}i.$$

where *T_nC_* and *T_nA_* are the effective transport numbers of ion *n* in the CEM and AEM, respectively, (n=1,2). The ion transport number in a membrane is defined as the fraction of the conduction current carried by ions of this species, for example, the transport number of ion *n* in the CEM is equal to *T_nC_ = j_n_z_n_/i* [20]. Since the conduction current, *i_F_*, is carried out by ions of both types, the following relations are fulfilled for membranes: *T*_1*C*_ + *T*_2*C*_ = 1, *T*_1*A*_ + *T*_2*A*_ = 1. In ion-exchange membranes placed in dilute electrolyte solutions, the current is carried almost only by counterions, that is, *T*_1*C*_ and *T*_2*A*_ are close to 1 [20].

The system of Equations (1)–(4) includes the electric field potential only in the form of spatial derivatives; therefore, only the potential drop, ϕ(h,t)−ϕ(0,t), is significant. For the convenience of calculations, we put the zero potential on the left boundary, *x = 0*:

(9) ϕ(0,t)=0.

On the other boundary, *x = h*, we use the condition that determines the derivative of the electric potential through the current density [21]:

(10) $$\frac{\partial \phi }{\partial x}\left(h,t\right)=-\frac{RT}{F^{2}}\left(\frac{i+\varepsilon \frac{\partial^{2}\phi }{\partial x\partial t}+Fz_{1}D_{1}\frac{\partial c_{1}}{\partial x}+Fz_{2}D_{2}\frac{\partial c_{2}}{\partial x}}{z_{1}^{2}D_{1}c_{1}+z_{2}^{2}D_{2}c_{2}}\right)\left(h,t\right).$$

At the initial moment, *t* = 0, the electroneutrality condition is satisfied at all points of the channel section, and the concentrations of cation and anion are equal to the initial concentration of the electrolyte, $c_{0}$; the potential is zero everywhere:

(11) $$c_{1}\left(x,0\right)=c_{0},\;\;c_{2}\left(x,0\right)=c_{0},\;\;\phi \left(x,0\right)=0.$$

### 2.2. System Parameters

The described above model (in Section 2.1) is a complex computational problem, since it involves a numerical solution simultaneously in a macroscopic (the channel width can reach several millimeters) and in a microscopic (the thickness of the quasi-equilibrium part of the SCR, λ, can be of the order of ten nanometers) regions. The fields of concentrations and potential in the quasi-equilibrium part of the SCR are characterized by huge gradients (Figure 1), which increase with decreasing thickness λ. The thickness λ is inversely proportional to the value $\sqrt{c_{0}}$ [22]; therefore, an increase in the electrolyte concentration, *c*_0_, significantly increases the computational complexity of the boundary value problem of the model. To obtain an acceptable accuracy of the results, the calculations in this section are performed for a low input concentration of the NaCl electrolyte solution, *c*_0_ = 0.001 mol/m^3^. Section 3.3 describes the calculations for a larger input electrolyte concentration (*c*_0_ = 2 mol/m^3^).

Values of other system parameters: the channel width *h =* 10^−3^ m; the system temperature *T* = 298 K; the diffusion coefficients of cations *D*_1_ = 1.33·10^−9^ m^2^/s and anions *D*_2_ = 2.04·10^−9^ m^2^/s; the charge numbers of ions *z*_1_ = 1, *z*_2_ = −1; *N_c_* = *N*_a_ = 1; water dielectric permittivity *ε_r_* = 81. The calculations are performed for two types of membranes: (a) perfectly selective membranes, that is *T*_2*C*_ = 0 and *T*_1*A*_ = 0; (b) membranes, the transport numbers of which correspond to the values of MK-40 [23], *T*_2*C*_ = 0.03 and *T*_1*A*_ = 0.03.

In the analysis of the study data of processes in membrane systems, it is accepted to normalize the current density by the value of the diffusion limiting current density. The value of the limiting current density can be determined by the Peerce formula [13]:

(12) $$i{\frac{FDc_{0}}{\delta \left(T_{1C}-t_{1}\right)}}_{lim},$$

where $D=D_{1}D_{2}\left(z_{1}-z_{2}\right)/\left(D_{1}z_{1}-D_{2}z_{2}\right)$ is the electrolyte diffusion coefficient, δ is the thickness of the diffusion layer, *t*_1_ is the cation transport number in the electrolyte solution.

Although there is no point in the concept of the diffusion layer and limiting current density in the system without the forced flow, we use the normalized current density *i/i*_lim_. The current density normalization facilitates the study of the similarity of transport processes at different electrolyte concentrations (as detailed in Section 3.3). When the diffusion layer thickness is $\delta =1.36\times 10^{-4}$m, the limiting current density is equal to $i_{lim}$=0.0019 A/m^2^.

The numerical solution of problem (1)–(11) was found by the finite element method on the non-uniform computational grid (the density of grid elements was increased near the solution/membrane interfaces). The implementation of the equation system is described in [21].

The error in the calculations performed for these parameter values, estimated as the difference between the average total current density in the channel, $1/h\int_{0}^{h}\left(i_{F}+i_{c}\right)dx$, and the specified current density, *i*, is less than 3%.

## 3. Results and Discussion

### 3.1. Space-Charge Breakdown

**Figure 2:**
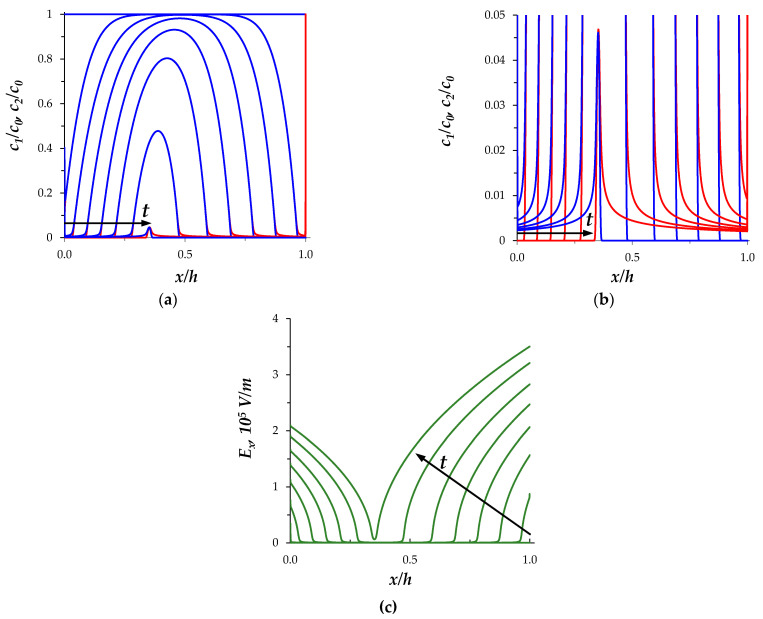
(**a**) Distributions of the normalized concentrations of cation, *c*_1_/*c*_0_, (red lines) and anion, *c*_1_/*c*_0_, (blue lines); (**b**) enlargement fragment of (**a**); (**c**) distribution of the electric field strength, E_x_. Calculation results at t = 0, 4, …, 24, 25.65 s for perfectly selective membranes *T*_2C_ = *T*_1A_ = 0, current density *i* = 2*i*_lim_.

**Figure 3:**
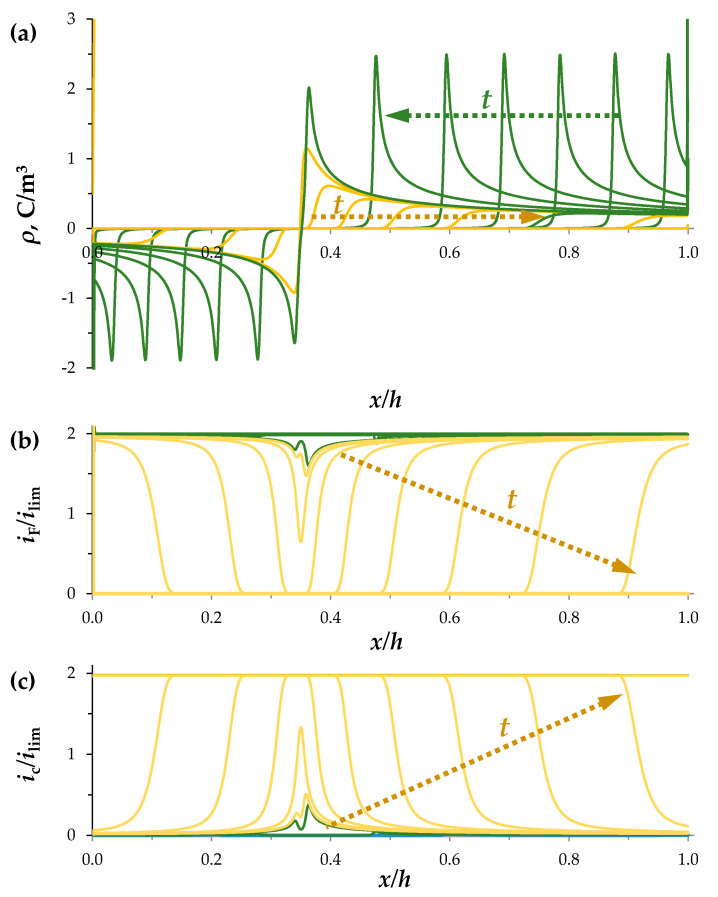
Distribution of the space charge density, *ρ*, (**a**), normalized conduction current density, *i_F_/i*_lim_, (**b**) and charging current density, *i_C_/i*_lim_, (**c**) at *t* = 0, 4, …, 24, 25.65 s (green lines) and during the discharge process *t* = 25.66, 25.67, …, 25.75 s (yellow lines) for the perfectly selective membranes, *T*_2C_ = *T*_1A_ = 0, *i* = 2*i*_lim_.

Consider the changes in the SCRs in the section of the desalination channel when the constant current density flows (*i* = 2*i*_lim_). Figure 2 shows the concentration profiles at times *t* = 0, 4, …, 24, 25.65 s, calculated for the perfectly selective membranes (*T*_2*C*_ = 0, *T*_1*A*_ = 0). As the current flows in the quiescent electrolyte layer, the concentrations of ions of both types decrease over time (Figure 2). In the regions near the membrane surfaces, desalination occurs faster, at $t_{C}\;$= 2.7 s an extended SCR appears at the CEM; later at $t_{A}$ = 6 s, at the AEM. Local extrema appear on the graph of the charge density, $\rho =F\left(z_{1}c_{1}+z_{2}c_{2}\right)$: a minimum is formed near the AEM surface and a maximum near the CEM (Figure 3a). In the SCRs, the conduction current, $i_{F}$, decreases, while the charging current, *i_c_*, increases (Figure 3b,c). The total current density, *i*, is constant, therefore, from Equation (4) it follows that the time derivatives of the charging and conduction currents have opposite signs, that is, the decrease in the conduction current is accompanied by an increase in the charging current. The electric field strength, $E_{x}=-\partial \phi /\partial x$, is low in the electroneutral region of the channel and significantly increases in the SCRs (Figure 2c).

The described behavior of the fields of ion concentrations, electric field strength, and current density components qualitatively coincides with the results of [6,18,21], taking into account the fact that these studies consider the diffusion layer.

Over time, the SCRs extend and their local extrema shift into the depth of the solution (green lines in Figure 3). At t≈26.65 s, when the electroneutral layer between the SCRs of opposite signs disappears, the space-charge breakdown occurs. The charge density, *ρ*, rapidly decreases and practically disappears in 0.1 s in the entire channel cross section (with the exception of quasi-equilibrium regions near the ion-exchange membranes), while the conduction current, $i_{F}$, decreases to 0, and the charging current, $i_{c}$, reaches the specified value of the current density, *i* (yellow lines in Figure 3).

The calculations of the ion concentrations and the electric potential based on the proposed model showed that the breakdown leads to an almost complete discharge of the space charge in the entire cross section of the desalination channel, except for the quasi-equilibrium SCRs near the membranes. Thus, the space-charge breakdown in the galvanostatic mode follows a scenario similar to that for the potentiostatic and potentiodynamic modes [15,16].

**Figure 4:**
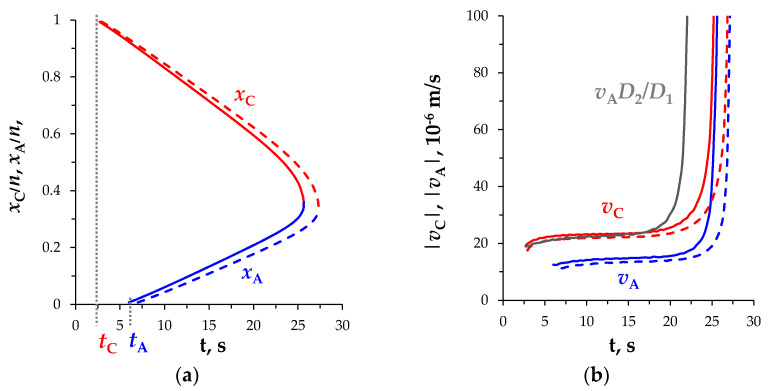
(**a**) Coordinates of the local extrema, *x*_C_ and *x*_A_; (**b**) Absolute values of the local extrema velocities, *v*_C_ and *v*_A_. The results for the space-charge extremum for the anion-exchange membrane (AEM) are marked in blue color, for the cation-exchange membrane (CEM)—in red color; *t*_A_ and *t*_C_ denote the moments of the appearance of the SCR extrema at the AEM and CEM, respectively; *T*_2C_ = *T*_1A_ = 0 (solid lines) and *T*_2C_ = *T*_1A_ = 0.03 (dashed lines), current density *i* = 2*i*_lim_. Value *v*_A_(*t*−*t*_A_)*D*_2_/*D*_1_ is shown in black in Figure (**b**).

In the considered case of the NaCl solution, the diffusion coefficients of ions are significantly different ($D_{2}/D_{1}\approx 1.53$); therefore, the extension of the SCRs near the CEM and AEM occurs at different rates. Figure 4 shows the results of calculating the coordinates of the local extrema of the SCRs at the CEM (*x*_C_) and AEM (*x*_A_) and the absolute values of their movement velocity, that is, the velocities of the SCRs extension, *v*_C_, *v*_A_. Once the SCRs are formed, the speed of movement of their local extrema increases slowly and at *t* > 25 s, when the distance between them becomes small (Figure 4a), the Coulomb forces of attraction significantly accelerate this process (Figure 4b). The velocity of the extremum of the space charge formed at the CEM, *v*_C_, is higher than that formed at the AEM, *v*_A_: the ratio of velocities of local extrema at equal intervals from their occurrence is approximately equal to the ratio of the diffusion coefficients, $v_{C}\left(t-t_{C}\right)/v_{A}\left(t-t_{A}\right)\approx D_{2}/D_{1}.$ The black line in Figure 4b represents the value $v_{A}\left(t-t_{A}\right)\cdot D_{2}/D_{1}$, which differs from $v_{C}\left(t-t_{C}\right)$ less than 6% from the moment the extremum appears to the area of a sharp velocity increase, that is, at *t* < 18 s.

Figure 4 also shows graphs for the case of the imperfectly selective membranes, *T*_2C_ = *T*_1A_ = 0.03 (dashed lines): the higher values of the effective transfer numbers of coions in membranes correspond to the lower velocity of the local extrema of the SCRs, since a certain additional number of coions enter the channel through the membranes.

### 3.2. Estimation of the Space-Charge Breakdown Time

In the galvanostatic mode, the space-charge breakdown time can be estimated from the current density set in the system. Before the start of the process of space-charge discharging, the value of the charging current density, *i_C_*, is small, as in the calculation shown in Figure 3, *i_C_* < 0.05*i*_lim_ at *t* < 25 s. Therefore, it can be assumed that the electric current in the electrolyte solution of the system under consideration is determined by the cation and anion fluxes formed during the complete electrolytic dissociation of the NaCl salt with the concentration *c*_0_, which was present in the channel section at the initial time (Equations (11)), $i\approx i_{F}\approx F\left(z_{1}j_{1}+z_{2}j_{2}\right)$. One should also take into account the coion fluxes into the desalination channel from adjacent chambers, which are associated with the imperfect selectivity of the membranes. When the transport numbers of ions in membranes are known, it is convenient to determine the current density at the membrane/solution interface. Thus, at the solution/CEM interface, *x = h*, the current density is equal to:

(13) $$i\approx \frac{z_{1}Fc_{0}h}{\tau_{b}}+T_{1A}i+T_{2C}i,$$

In Equation (13), the first term is the current density of the solution cations. If we define the time of the space-charge breakdown, $\tau_{b}$, as the time of the complete ion depletion, then this current is equal to $z_{1}Fc_{0}h/\tau_{b}$. The second term is the current of cations entering the channel through the AEM; the third term is the current of anions entering the channel through the CEM.

From Equation (13), it is easy to obtain a formula for the time of the space-charge breakdown, taking into account the selective properties of the membrane:

(14) $$\tau_{b}\approx \frac{z_{1}Fc_{0}h}{i\left(1-T_{1A}-T_{2C}\right)}.$$

A similar expression can be written for the AEM/solution interface.

Equation (14) shows that, in the galvanostatic mode, the time of the space-charge breakdown (in the dimensional form) is directly proportional to the electrolyte concentration at the initial time and the channel width, and is inversely proportional to the current density. Thus, the space-charge breakdown is a limitation of the possibility of increasing the mass transfer efficiency in electromembrane systems by reducing the intermembrane distance (the channel width) or increasing the current density. Equation (14) also reflects the fact that an increase in the transport numbers of coions in the membrane increases the breakdown time. In the limiting case, when membranes are equally permeable to ions of both types (cations and anions), *T*_2C_ = *T*_1A_ = 0.5, the complete desalination of the solution will never occur, $\tau_{b}\to \infty$.

**Figure 5:**
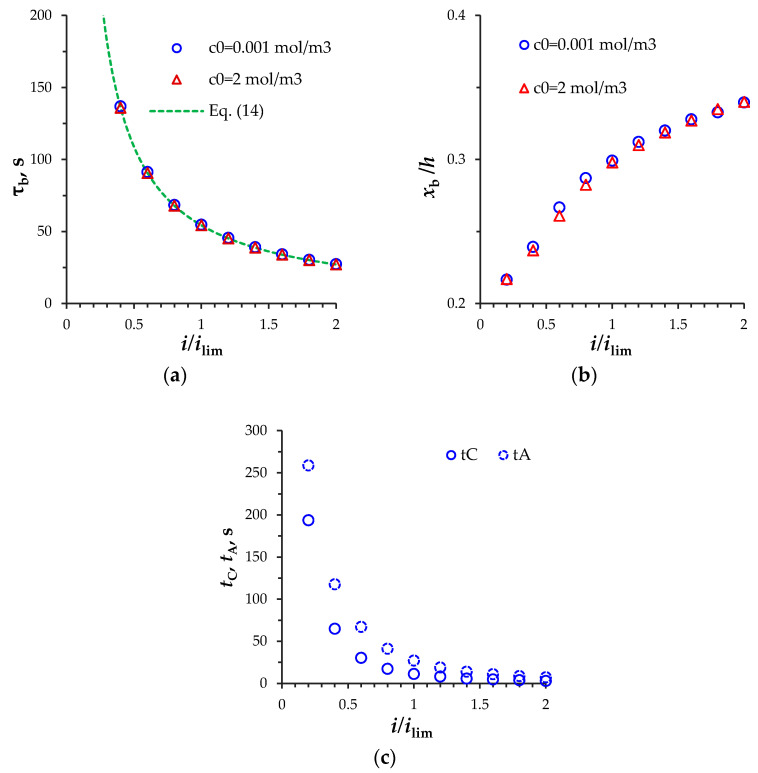
(**a**) The space-charge breakdown time, *τ_b_* calculated by the model (markers) and by Equation (14) (dashed line); (**b**) distance from the AEM to the local minimum of the SCR before the discharge, $x_{b}$; (**c**) the time of the appearance of local extrema of the SCRs at the AEM, *t_A_*, and CEM, *t_C_*. Results for *T*_2C_ = *T*_1A_ = 0.03, *c*_0_ = 0.001 mol/m^3^ (round markers) and *c*_0_ = 2 mol/m^3^ (triangular markers).

Figure 5a shows the dependences of the breakdown time on the current density, calculated by Equation (14) and by the model as the moments when the extended SCRs disappear completely. The difference between these calculations of $\tau_{b}$ at the current density from 0.1*i*_lim_ to 2*i*_lim_ does not exceed 1%.

The current density also affects the position of the local extrema of the SCRs before the discharge: the greater the current density, the greater the distance from the AEM to the local minimum of the SCR before the discharge, $x_{b}$, (Figure 5b). This is due to the fact that with an increase in the current density, the time of the SCR appearance at the AEM, *t_A_*, decreases faster than at the CEM, *t_C_*, (Figure 5c).

### 3.3. Simplification of the Model

To simplify the calculations and obtain results for a larger input electrolyte concentration, we will use the method of excluding the quasi-equilibrium part of the SCR from the consideration [21], according to which boundary conditions (5) and (6) are replaced by conditions (15) and (16):

(15) $$\frac{\partial c_{1}}{\partial x}\left(h,t\right)=0,$$

(16) $$\frac{\partial c_{2}}{\partial x}\left(0,t\right)=0.$$

**Figure 6:**
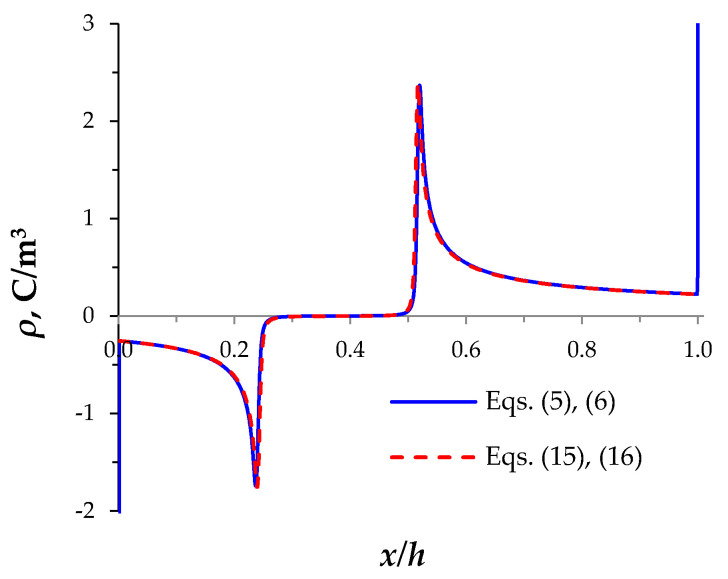
Distribution of the space charge density, *𝜌*, at *t* = 24 s calculated with Equations (5) and (6) (solid line) or with Equations (15) and (16) (dashed line) for *c*_0_ = 0.001 mol/m^3^, *T*_2C_ = *T*_1A_ = 0.03, *i* = 2*i*_lim_.

The calculation results with Equations (5) and (6) and with Equations (15) and (16) are close to each other everywhere except for the quasi-equilibrium parts of the SCR (Figure 6). The use of Equations (15) and (16) instead of Equations (5) and (6) leads to a slight overestimation of the SCR extension velocities (Figure 6). The difference in the breakdown time for these two variants of setting the boundary conditions does not exceed 1% for *c*_0_ = 0.001 mol/m^3^, *T*_2C_ = *T*_1A_ = 0.03, at $i=0.2i_{lim},\;\ldots ,\;2i_{lim}$.

Conditions (15) and (16) made it possible to calculate the model for the electrolyte concentration *c*_0_ = 2 mol/m^3^ with sufficient accuracy (the difference between the average total current density in the channel, $1/h\int_{0}^{h}\left(i_{F}+i_{c}\right)dx$, and the specified current density, *i*, is less than 0.03%). The results of this calculation are shown by triangular markers in Figure 5a,b. Figure 5 shows that, at the normalized current density, the electrolyte concentration, *c*_0_, does not affect the time, $t_{b}$, and distance from the AEM to the local minimum of the SCR before the discharge, $x_{b}$: the difference in calculations of the time $\tau_{b}$ for *c*_0_ = 0.001 mol/m^3^ and 2 mol/m^3^ is less than 1% and in calculations of $x_{b}$ is less than 2.2%. Indeed, if the current density *i*/*i*_lim_ (normalized using Equation (12)) is introduced into Equation (14), the breakdown time, $\tau_{b}$, will not depend on the ion concentration, *c*_0_.

Thus, calculations with Equations (15) and (16) have an acceptable accuracy and allow calculations for electrolyte concentration values corresponding to those used in actual experiments and in practice.

## 4. Conclusions

The desalination of the electrolyte solution by intense currents in the section of the membrane channel in the galvanostatic mode are investigated through the developed numerical model. The model describes the SCRs changes from formation at the surfaces of the ion-exchange membranes to complete discharge. The simplification of the model by excluding a quasi-equilibrium part of the SCR from the consideration made it possible to obtain the numerical solution for the values of the electrolyte concentration used in experiments. Numerical results have shown that the ratio of the ion diffusion coefficients determines the ratio of the SCR extension velocities of different signs and the location of the space-charge breakdown, but does not affect the breakdown time. An approximate formula is derived to estimate the space-charge breakdown time for the galvanostatic mode, taking into account the selective properties of the membrane. The difference between the breakdown time calculated by using the approximate formula and the proposed model does not exceed 1%. It is shown that the deviation of the selective properties of the membrane from the perfect values leads to the slowdown in the processes of desalination and the delay in the onset of the space-charge breakdown.

The development of the proposed model for the 2D case will make it possible to study the phenomenon of space-charge breakdown in the desalination channel at a direct current, taking into account the forced flow and the development of electroconvection. Dissociation/recombination reactions of water molecules are another aspect of further investigation of the space-charge breakdown.

## Data Availability

Not applicable.
